# Bioprinted High-Cell-Density Laminar Scaffolds Stimulate Extracellular Matrix Production in Osteochondral Co-Cultures

**DOI:** 10.3390/ijms252011131

**Published:** 2024-10-17

**Authors:** Aidan Bowes, Amy Collins, Fiona Oakley, Piergiorgio Gentile, Ana Marina Ferreira, Kenny Dalgarno

**Affiliations:** 1 School of Engineering, Newcastle University, Newcastle upon Tyne NE1 7RU, UK; aidan.bowes@sunderland.ac.uk (A.B.); piergiorgio.gentile@newcastle.ac.uk (P.G.); ana.ferreira-duarte@newcastle.ac.uk (A.M.F.); 2 Faculty of Technology, Sunderland University, Sunderland SR6 0DD, UK; 3 Newcastle Fibrosis Research Group, Bioscience Institute, Faculty of Medical Sciences, Newcastle University, Newcastle Upon Tyne NE2 4HH, UK; amy.collins2@newcastle.ac.uk (A.C.); fiona.oakley@newcastle.ac.uk (F.O.)

**Keywords:** biphasic scaffold, collagen–alginate–fibrin hydrogel, bioprinting, osteochondral model, co-culture, in vitro ECM production, cell density

## Abstract

Many tissues have a laminar structure, but there are limited technologies for establishing laminar co-cultures for in vitro testing. Here, we demonstrate that collagen–alginate–fibrin (CAF) hydrogel scaffolds produced using the reactive jet impingement bioprinting technique can produce osteochondral laminar co-cultures with well-defined interfaces between cell types and high cell densities to support cell–cell interaction across the interfaces. The influence of cell density and the presence of the two cell types on the production of extracellular matrix (ECM) and the emergent mechanical properties of gels is investigated using IHC, ELISA, gel mass, and the compression modulus. The results indicate that high-cell-density cultures and co-cultures with these specific cell types produce greater levels of ECM and a more biomimetic in vitro culture than low-cell-density cultures. In laminar scaffolds produced using TC28a2 chondrocytes and SaoS-2 osteoblasts, both cell density and the presence of the two cell types enhance ECM production and the mechanical properties of the cultures, presenting a promising approach for the production of more biomimetic in vitro models.

## 1. Introduction

There is currently no generally accepted in vitro model of the bone–cartilage interface which can serve as a basis for disease modelling or drug testing for osteoarthritis (OA) or other diseases which affect joint surfaces [1,2]. Pharmacological and regenerative treatments for OA are the subject of many commercial and academic research programmes, but the lack of effective models of joint interfaces is a significant bottleneck in the development of new treatments. Whilst in vivo animal studies are often more representative, there are other factors to consider that limit their use, including cost, the relatively low throughput, and the desire to move towards non-animal technologies [3,4]. Validated and high throughput human in vitro models of OA would provide a step change in drug development for this debilitating disease.

Research into the development of in vitro osteochondral interface models has considered a wide range of strategies, with the need for the bone and cartilage tissue models to be in close proximity considered essential [5]. Many studies involve researchers manually seeding cells on to a porous scaffold [6,7,8] or using transwell co-cultures [9], but more recently, there have been attempts to produce layered osteochondral scaffolds with cells encapsulated within the inks in order to forgo the additional step of cell seeding. A 3D hydrogel environment has been shown to be useful for matrix production [10], and Kilian et al. [11] used extrusion based printing to produce multi-layered osteochondral tissue models, building the model using calcium phosphate cement and a bioink based on alginate–methylcellulose. These models produced ECM but the cell viability was low (<60%), with the high viscosity bioink causing high shear stresses during extrusion and damaging the cells [11]. Chondrocyte laden constructs for cartilage tissue engineering applications have also been produced using lower viscosity inks based on nanocellulose–alginate [12] (viability~70%) and gelatin-methacrylamide (viability~80%) [13]; however, the latter did not allow for the production of volumetric constructs without support. The use of 3D meshes as a way of representing different zones in multi-layer scaffolds is common, with more dense zones being represented by low-porosity/high-stiffness structures and less dense zones represented with higher-porosity/lower-stiffness structures. A wide range of fabrication techniques have been employed, including wet spinning [14], melt electro-writing [15,16], and fused filament fabrication [17,18]. However, there are limitations to this approach as low-porosity scaffolds can be difficult to seed and they limit the perfusion of oxygen and nutrients to seeded cells, and the approaches are complex, often involving the assembly of multiple scaffolds.

In the development of in vitro tissue models and tissue engineering strategies more generally, the importance of cell density and 3D architecture is well recognised. Cell–cell interactions and communication are an important component of recapitulating physiological behaviour, and high-cell-density 3D structures offer a biomimetic architecture for this [19,20,21]. This has led to significant interest in cell spheroids across a range of tissue types. For cartilage in particular, cell spheroids are exploited for tissue engineering [22,23,24] and for in vitro models [25], and this can be extended to spheroids with both chondrocytes and mesenchymal stem cells [26]. However, cell spheroids do have limitations, as they can take a long time to form, and the ease with which they can be formed is dependent on the type of cell or cells of interest [27,28]. The “scaffold-free” nature of spheroids is appropriate for some cell types, but it is less valuable for other cell types where the initial attachment to ECM or an ECM analogue is a key part of the process. In the context of an osteochondral model, mineralisation is a key process in the formation of bone neo-tissue and requires osteoblasts to be attached, and this is enhanced by the availability of a scaffold. For bone tissue models, the quality of the neo-tissue produced and the rate at which the models mature are also known to be dependent on the initial cell density [29]. Many osteochondral tissue engineering strategies have developed laminar structures, encompassing a cartilage layer, a subchondral bone layer, and sometimes an intermediate layer representative of calcified cartilage [30]. There is also evidence that cross-talk between bone and cartilage cells influences homeostasis in healthy tissue and disease progression in osteoarthritic tissue [31], but the effect of this has not to date been explored in the in vitro evaluation of osteochondral tissue engineering strategies, and the need for an in-depth understanding of the effects of co-cultures for cartilage tissue engineering is well recognised [32]. The aim of this study was to evaluate the effect of co-cultures and cell density on the initial formation of laminar osteochondral co-cultures to inform the development of osteochondral models.

The bone cartilage interface is essentially a laminar structure, with a layer of cartilage over the top of subchondral bone, and so scaffolds which have a laminar structure are more physiologically representative than spheroid cultures. Here, we present a systematic study on the initial formation of laminar osteochondral co-cultures in vitro. To produce cells within a laminar scaffold, we printed cells encapsulated within a hydrogel matrix using the reactive jet impingement (ReJI) bioprinting process [29,33]. ReJI bioprinting can produce high-cell-density gels, and to print on a wide range of substrates [34,35,36], including printing gels on gels. These two capabilities give high-cell-density scaffolds the potential to be created with distinct zones of different cell types but with close cell contact at the interfaces between those zones. Osteochondral co-cultures allow osteoblasts and chondrocytes to interact, and so we compared the development of chondrocyte and osteoblast single cell cultures with osteochondral co-cultures and also explored the influence of starting cell density on how all three of the culture types mature. This allowed for an in-depth study of the trade-off between the gel being digested by cells and the extracellular matrix being produced by the cells, and we show that in the development of all three culture types, the starting cell density plays a critical role in defining mechanical properties via ECM production, and for the co-culture, the presence of the two cell types stimulates further ECM production and further enhances the mechanical properties of the cultures. This demonstrates that high-cell-density gel-based co-cultures offer the cell–cell and cell–ECM interactions required to produce representative tissues for disease models and drug testing.

## 2. Results and Discussion

### 2.1. Scaffold Production Using ReJI Bioprinting

**Figure 1:**
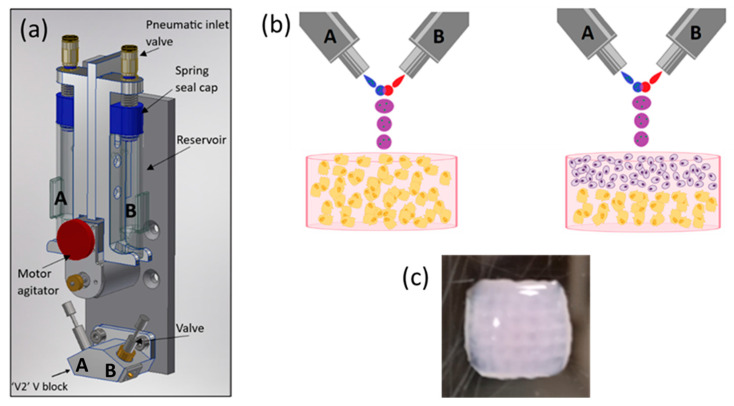
(**a**) A schematic CAD model showing the arrangement of a bio-ink reservoir and microvalves within a ReJI printhead. Tubes directly link the reservoirs to the microvalves so that reservoirs A and B feed valves A and B respectively. (**b**) The microvalves produce droplet streams which collide and react to produce gel droplets which fall to the substrate to create monoculture gels (**left**, osteoblasts in gel) or, with sequential printing, co-culture gels (**right**, chondrocytes and osteoblasts or MSCs in laminar culture). (**c**) An example printed hydrogel viewed from above, 6 mm square, 3 mm thick. (**b**) created with BioRender.com.

The schematics of an ReJI printhead and the structure of printed monoculture and co-culture gels are shown in Figure 1. The process involves creating droplet streams of a polymer solution (from valve B, Figure 1b) and a solution which induces crosslinking (from valve A). The droplet streams meet in mid-air and react to form a gel droplet which falls to the substrate. If a cell-filled gel is desired, cells are initially suspended in one or both of the starting solutions, and in the work presented here, cells were suspended in the solution which induced crosslinking.

The printed gel cultures were produced in a collagen, alginate, fibrin (CAF) hydrogel, which provides an excellent environment for cell cultures [37]. Gels were printed into CELLSTAR^®^ flat-bottom 96-multiwell plates, and the printed gels were 7 mm in diameter and 3 mm in height. When creating stratified co-cultures, first a half-height (1.5 mm) volume of gel containing osteoblasts was printed, then a second half-height volume containing chondrocytes was printed on top to attain a total gel thickness of 3 mm, as shown in Figure 1.

Printed gels were transferred to 24-multiwell plates and cultured in 2 mL of the relevant cell culture media. For the co-culture gels, a 1:1 blend of the osteoblast/chondrocyte cell culture media was used. The culture conditions were 37 °C, 20% O_2_ and 5% CO_2_ in 24-well plates, with supernatant removed and replaced with fresh culture media every 24 h.

**Figure 2:**
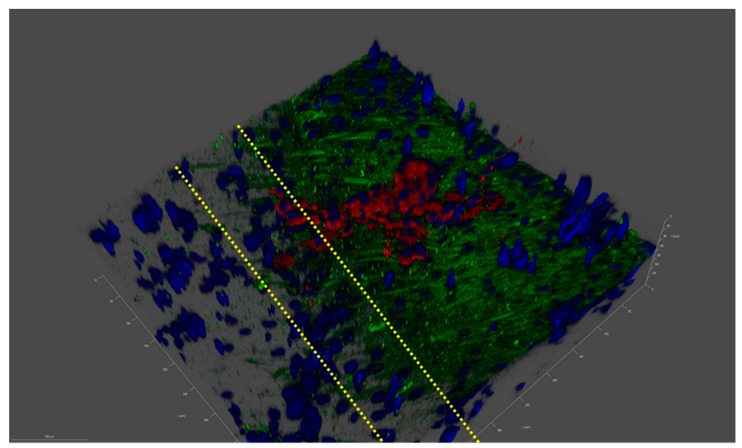
A confocal image of the MSC–chondrocyte co-culture proof-of-concept study showing a laminar structure. An imaged interface between a printed layer of Y201 MSC cells and TC28a2 chondrocyte cells after 3 days of co-culture in F12/DMEM media. Blue: cell nuclei; red: aggrecan; and green: collagen II. Dashed yellow lines identify the laminar interface, which is a few cells thick, through the change in collagen II expression as we move from Y201 cells to TC28a2s. Tick marks on the scale are 10 µm apart.

**Figure 3:**
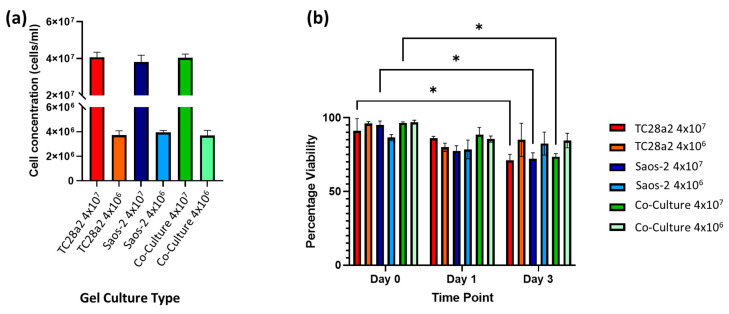
(**a**) The cell concentration (cells per mL) of cells in digested hydrogels immediately after printing (n = 3). (**b**) The post-print viability of cell-filled 3D-printed hydrogels, containing TC28a2, Saos-2, and TC28a2/Saos-2 co-cultures (n = 2). There is no significant difference between the viability of the different cell types in any of the printed densities at any of the timepoints. When *p* ≤ 0.05 is represented by *, and *p* > 0.05 is not significant and not represented on the graph.

A preliminary study with ReJI-bioprinted MSC and chondrocyte co-cultures was carried out to assess the interface produced when printing. Figure 2 shows that the two gel cultures form a tight and well-defined interface which is maintained through the initial culture. With this established, further analysis focused on osteochondral cultures, and to establish an osteochondral structure from day 0, osteoblasts were used together with chondrocytes in preference to MSCs. The ReJI bioprinting system was able to deposit osteochondral gels with accurate cell densities, and the post-print viability of cultures and co-cultures printed at densities of 4 × 10^6^ cells/mL and 4 × 10^7^ cells/mL was high (Figure 3). The overall cell viability was slightly lower for the high-cell-density co-cultures than for the low-cell-density co-cultures at day 3, most likely reflecting the greater competition between cells for nutrients within the gel.

### 2.2. ECM Production

**Figure 4:**
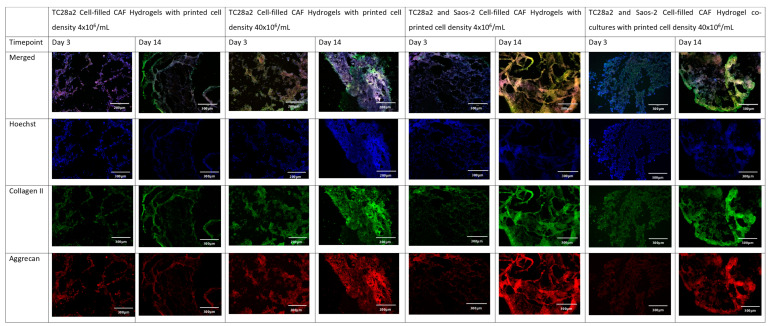
The immunohistochemical staining of the chondrocyte region of the co-culture. Sections stained to show the presence of cell nuclei (blue), collagen II (green), and aggrecan (red).

**Figure 5:**
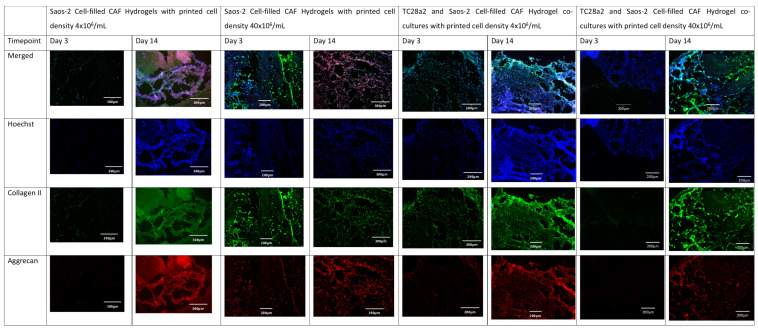
The immunohistochemical staining of the osteoblast region of the co-culture. Sections were stained to show the presence of cell nuclei (blue), osteocalcin (green), and osteopontin (red).

Collagen II and aggrecan are recognised biomarkers for ECM development in cartilage [38], and osteopontin, osteocalcin [39], and collagen I [40] are indicative of bone ECM formation. All samples (Figure 4 and Figure 5) showed evidence of either cartilage or bone ECM markers at day 3, indicating that the bioprinting approach did not inhibit functionality, although for the high-density co-culture, the expression of both cartilage and bone biomarkers at day 3 was quite limited, suggesting that these cultures took more time to establish. The expression of bone and cartilage markers at day 14 is generally greater than that at day 3, so all of the cultures continue to mature over the culture period. Despite the limited ECM production at day 3, at day 14, the co-culture samples have a higher concentration of cartilage and bone markers than the corresponding single cell-type culture, indicating that over the time period as a whole, cells within the co-cultures produce more ECM. It is notable that aggrecan production in a chondrocyte-laden hydrogel is often not observed until much later timepoints. For instance, Skaalure et al. [41] in their study of aggrecan production in chondrocytes in PEG hydrogels seeded a similar density of cells (5 × 10^7^ cells/mL) manually on to gels, but they needed six weeks of culture to produce similar levels of aggrecan to those seen here at day 14. In addition, the monoculture high-density gels generally produced more ECM than the low-density gels, with this especially notable at day 3, reinforcing the hypothesis that greater cell–cell and cell–ECM production positively affects cell functionality and produces cultures that mature quicker [42,43,44]. Within gel cultures with proliferative cells, we generally observe that proliferation rates are higher in low-cell-density gels than in higher-density ones so that cell densities become closer over time [35]. In essence, this means that while having a high initial cell density with proliferative cells saves time, over a long culture period, cells will eventually proliferate to the stage where the cell densities are the same. However, in cultures where non-proliferative cells are used, differences in the initial cell number will not be bridged over time, meaning that high-cell-density cultures will always offer greater cell–cell interaction.

No large volumes of cell necrosis were observed, despite the large size of the starting gel cultures. This result aligns with previous work with ReJI-printed CAF gels, with sustained cell viability in gels for culture periods of up to 21 days [34,35,36], arising from the organisation of cells within the gels supporting diffusion. At later timepoints, cells appear to be linked in strands, with web-like formations containing large areas of visible porosity which would support oxygen and nutrient diffusion.

**Figure 6:**
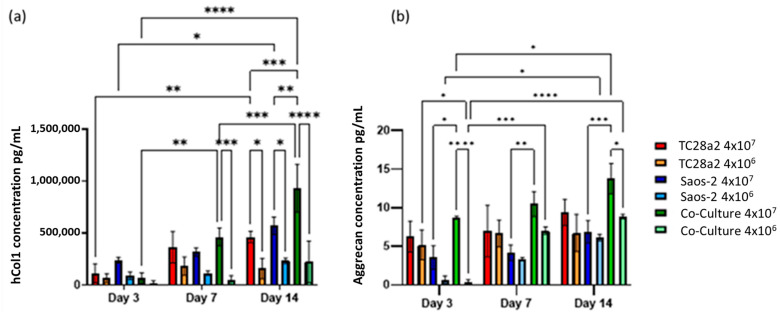
(**a**) Human collagen I and (**b**) aggrecan concentration in supernatant removed from chondrocyte, osteoblast, and co-cultures, with cell densities of 4 × 10^7^ cells/mL (high density) and 4 × 10^6^ cells/mL (low density). n = 3. At day 14, the high-density co-culture showed significantly higher concentrations of hCol1 than all other high- and low-cell-density cultures. At all timepoints, the average aggrecan concentration in the high-density cultures is higher than that in the corresponding low-density cultures, although this difference is only significant in the co-culture samples. The level of aggrecan detected in the high-density co-culture samples is significantly higher than that in the corresponding low-density samples at both days 1 and 14. When *p* ≤ 0.05 is represented by *, *p* ≤ 0.01 is **, *p* ≤ 0.001 is ***, *p* ≤ 0.0001 is ****, and *p* > 0.05 is not significant and not represented on the graph.

Greater concentrations of collagen I and aggrecan were seen in high-cell-density cultures rather than low-cell-density cultures (Figure 6), with this particularly notable for collagen. For both proteins, the high-density co-culture shows the highest average levels of protein concentration across five of the six timepoints, however not always with significance at those timepoints.

Overall, we observe that as the cell density increases, so does the rate of ECM production, indicating that printing with the highest cell density is the quickest way to create biomimetic tissue models with these cell types. We also observed that the formation of an ECM in co-cultures is greater than that found in the single cell cultures, particularly evident for hCol1 in Figure 6a, but reinforced by Figure 4 and Figure 5. It is also clear for these cell types that growth in a co-culture has a large impact on aggrecan production as we obtain the surprising result that the aggrecan detected in the high density co-culture samples is significantly higher than the corresponding high density monoculture samples at day 14, even though the co-cultures have half the number of chondrocytes and half the number of osteoblasts of the equivalent cell density monocultures. This together with the observations on hCol1 suggests that both the TC28a2 chondrocytes and the Saos-2 osteoblasts are being stimulated to produce more ECM by the presence of the other cell type.

### 2.3. Mass and Mechanical Properties in Compression

**Figure 7:**
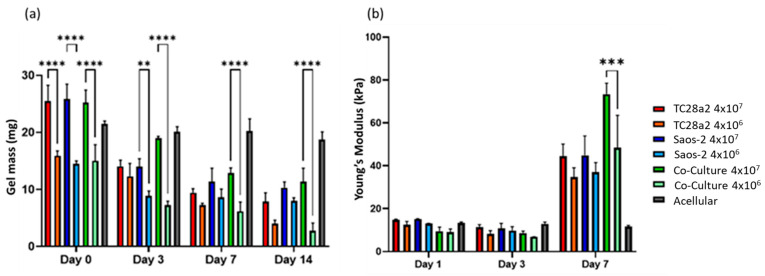
(**a**) The average gel mass and (**b**) compression modulus of gel cultures. For clarity, significance is illustrated only for high-density/low-density comparisons with a single culture type at each timepoint. n = 3. The compression modulus did not significantly increase for any of the cultures between days 1 and 3, but all cultures showed a significant increase in the modulus between days 3 and 7, with the high-cell-density co-culture showing the greatest increase. When *p* ≤ 0.01 is represented by **, *p* ≤ 0.001 is ***, *p* ≤ 0.0001 is ****, and *p* > 0.05 is not significant and not represented on the graph.

All the gel cultures lost mass over the 14-day culture period, Figure 7, with the high-cell-density gels starting out heavier and remaining heavier throughout the culture period. We consider that there are four competing mechanisms at play in determining the mass changes in the gels. The first is cell migration from the gels, the second is cell proliferation, the third is gels being digested by the cells [45], and the fourth is ECM production by the cells. Acellular gels exhibited no significant loss of mass over the entire two-week period, so gel dissolution in the absence of cells is not a factor. The digestion of the gels is related to cell migration through the gels (cells enzymatically degrade their surrounding matrix in order to create space to move into), so counter-intuitively, this can be initially higher in low-cell-density gels, where there is less competition for space and as a result more migration. The increase in the compression modulus between day 3 and day 7 for all the gels can only be explained through proliferation and ECM production. The fact that there was no increase between day 1 and day 3 suggests that proliferation and/or ECM production does not pick up significantly until day 3 or that gel digestion is concentrated in the first few days of culture. The fact that the co-culture shows a significantly larger modulus increase between day 3 and day 7 when compared to the single cell cultures is considered to further reinforce the IHC and ELISA results in terms of the co-culture demonstrating increased levels of protein production. The compressive modulus of the gels in absolute terms is below that reported for articular cartridge (240−1000 kPa [46]), and so future work could consider a revised gel formulation and printing onto harder substrates (as shown by Kotlarz et al. [34]) to create a more biomimetic bone–cartilage interface.

## 3. Materials and Methods

### 3.1. Cell Culture

To maintain consistency through the tissue models, two well-characterised cell lines were used to create the co-cultures: the TC28a2 chondrocyte cell line [47] and the osteoblastic Saos-2 cell line [48] (both obtained from Sigma Aldrich, Gillingham, UK).

The TC28a2 chondrocyte cell line and Y201 MSC cell line were cultured in Dulbecco’s Modified Eagle Medium/Nutrient Mixture F-12 (Gibco™ DMEM/F-12 high glucose; Fisher Scientific, Loughborough, UK) adding 10% FBS (Fetal Bovine Serum, Gibco, Fisher Scientific, Loughborough, UK) and 5000 Uml−1 penicillin–streptomycin (Sigma Aldrich, Gillingham, UK).

The Saos-2 osteosarcoma cell line was cultured in McCoy’s 5A (Modified) Medium (Fisher Scientific, Loughborough, UK) adding 20% FBS (Fetal Bovine Serum, Gibco, Fisher Scientific, Loughborough, UK) and 5000 Uml−1 penicillin–streptomycin (Sigma Aldrich, Gillingham, UK). Additionally, 20% FBS was used as it was found that this helped the cells to proliferate faster.

For TC28a2/Saos-2 co-cultures, mixed media were used. F12/DMEM and McCoy’s 5A are both general cell growth media, and it was shown that these can be used interchangeably between each of these cell types with no adverse effects [49,50]. As the cell mix ratio in printed co-cultures was 1:1, this was the same ratio used for the media combination.

Cells were cultured in a Thermo Forma incubator at 37 °C, 21% O_2_, and 5% CO_2_ in 175 cm^2^ (T175) Corning tissue culture flasks. When cells reached 75–85% confluence, they were passaged and split into separate T175 flasks with a seed density of 750,000–1 million cells per flask.

### 3.2. Gel Printing and Culture

The polymer solution (CAF precursor) comprises collagen (6 mg/mL Pepsin-Soluble Collagen in 0.01 M HCI, Collagen Solutions, Glasgow, UK), alginate (alginic acid sodium salt from brown algae, Sigma—180,947 at a concentration of 25 mg/mL in PBS), and fibrinogen (fibrinogen from bovine plasma, Sigma—F8630 at a concentration of 37 mg/mL in PBS) solutions mixed with a 1:2:8 ratio, respectively. The preparation process was firstly to gradually add sodium alginate to PBS held at 35 °C over a period of 30 min to 1.5 h (dependent on the amount of alginate added and the solution batch size) whilst stirring continually until dissolved. Fibrinogen was then added to PBS pre-warmed to 35 °C and stirred at this temperature for 30 min. The solution was then aspirated up and down with a 1 mL micro-pipette and filtered through a yellow Corning FalconTM cell strainer with a mesh size of 100 μm. Alginate was then added to the filtered fibrinogen working solution and aspirated up and down with a micro-pipette until fully mixed. The collagen solution was then added to this mixture. Finally, the CAF precursor was filtered through a yellow Corning FalconTM cell strainer with a mesh size of 100 μm. The crosslinking solution was produced by mixing 500 U of thrombin (T4648, Sigma) and 16 µL of 6% CaCl_2_ in 1 mL of high-glucose media. For acellular gels, the CAF precursor solution and crosslinking solutions were used to directly print gel samples. When printing cell-filled gels, the cells were first centrifuged, and the supernatant was removed. The cells were then re-suspended in the thrombin solution and at this point were ready for printing.

All printing was performed using the ReJI bioprinter head mounted to a JetLab^®^ 4 XL (MicroFab, Plano, TX, USA) printing work station combined with the JetDrive^®^ printer drive electronics unit. The valves used in the ReJI system are INKX0514950A VHS Solenoid valves supplied by Lee Valves (Gerrards Cross, UK). These were operated using a 24 V, 62 µs “spike” signal to open the valves, then a 5 V 738 µs “hold” signal to keep the valve open. The CAF precursor was printed at a pressure of 0.6 bar, and the thrombin solution was printed at a pressure of 0.5 bar, with 400 droplets printed per second.

### 3.3. Characterisation of Printed Gels

#### 3.3.1. Cell Viability

To digest gels, first, the supernatant was removed from the gels in the well plates, and the gels were washed three times with PBS. Gels were then removed from 24-well culture plates and placed in 48-well plates. A total of 0.5 mL of room temperature 1 U/mL dispase solution in DMEM/F12 (Stemcell Technologies, Cambridge, UK) was added to each well, and the well was placed on a Stuart SSM1 orbital plate shaker at 100 rpm for 5 min. Gels were then incubated at 37 °C for one hour and pipetted up and down three times. The pellet was then re-suspended in the LIVE/DEAD^®^ Viability/Cytotoxicity Kit for mammalian cells (Fisher Scientific, Loughborough, UK). Cells were imaged using a Tali cell counter with red and green filters compatible with the live/dead assay stainings. Three samples of each cell density and culture type were imaged at each timepoint. Imaging was performed on day 0, day 1, and day 3 following printing.

#### 3.3.2. ECM Production

At each relevant timepoint, gels were removed and placed in a 48-multiwell plate and washed twice with DPBS (Dulbecco’s Phosphate-Buffered Saline, Sigma—D8537); then, approx. 500 µL of 4% PFA (Paraformaldehyde Solution, 4% in PBS, Thermo Scientific™—AAJ19943K2) or enough to fully cover the sample was added to each well containing a sample and left overnight at 4 °C. PFA was then aspirated from the well plates, and samples were again washed with DPBS twice before being covered with DPBS.

Prior to staining, DPBS was removed, and all of the gels were blocked with 0.1% Triton X-100 for 20 min and then blocked with 3% goat serum at room temperature for 1 h and 15 min.

When staining for collagen markers, the gels were incubated overnight with primary antibodies at 4 °C against collagen II 1:200 (Collagen II Rabbit anti-Bovine, Human, Mouse, Ovine, Rat, Polyclonal, Invitrogen™, Fisher Scientific, Loughborough, UK—PA126206) and aggrecan 1:200 (Aggrecan Mouse anti-Bovine, Canine, Equine, Feline, Guinea Pig, Human, Ovine, Porcine, Rabbit, Rat, Clone: BC-3, Invitrogen™, Fisher Scientific, Loughborough, UK—11555772). For bone markers, the samples were incubated overnight with primary antibodies at 4 °C against Osteocalcin Antibody 1:100 (PA5-96529—Thermofisher) and Osteopontin Monoclonal Antibody 1:100 ((2F10), eBioscience™, Fisher Scientific, Loughborough, UK).

Gels were then incubated at room temperature with Goat anti-Mouse IgG (H + L) Highly Cross-Adsorbed Secondary Antibody, Alexa Fluor Plus 647 (Fisher Scientific, Loughborough, UK—A32728), and Goat anti-Rabbit IgG (H + L) Highly Cross-Adsorbed Secondary Antibody, Alexa Fluor Plus 488 (Fisher Scientific, Loughborough, UK—A32731), at a concentration of 1:200 diluted in DPBS for 45 min.

Finally, 1 µL of 0.1% Hoechst 33342 solution (Fisher Scientific, Loughborough, UK—62249) was added to each sample and incubated at room temperature for 15 min. Between each stage, the gels were washed three times with DPBS.

After removing DPBS, samples were taken to the Leica CM1590 cryostat for cryosectioning. A small amount of an OCT (optimal cutting temperature) compound was placed on the sample dies, which were pre-chilled to −20 °C. Once the OCT began to set slightly at the edges, the gel samples were placed in the middle and covered entirely in OCT. The dies were then returned to the −20 °C machine bed to fully freeze. Once fully frozen, the die with the sample was placed on the cryotome head, which was also set at −20 °C, and 20 μm slices were made which were collected on pre-coated slides. All samples were imaged using the EVOS M5000 fluorescence microscope with an Olympus 10*X* Aprochromat objective, using settings specific for Hoechst 33342, Alexa Fluor Plus 488, and Alexa Fluor Plus 647 using the following excitation and emission settings: Hoechst 33342, 357−444 nm excitation and 447−460 nm emission; Alexa Fluor Plus 488, 470−522 nm excitation and 525−550 nm emission; and Alexa Fluor Plus 647, 628−640 nm excitation and 685−740 nm emission.

#### 3.3.3. Confocal Microscopy

Cultures were imaged using the GaAsP-Pmt1 and GaAsP-Pmt2 detectors of the Zeiss LSM800 (Jena, Germany) in confocal mode, with a pinhole of 53 um diameter (1.68 AU) giving an optical section of 0.940 µm. The x/y sampling was set at 512 × 512 at a scan zoom of 0.5x (giving a scaling of 1.248 µm per pixel), and 251 z slices were taken over 235 µm to create the Z stack.

#### 3.3.4. ELISA

Protein production was measured quantitatively by analysing the media supernatant using human collagen I (HCol1) and aggrecan (PG) ELISAs. The HCol1 ELISA (Human Pro-Collagen I alpha 1 DuoSet ELISA—DY6220-05) was performed as per the manufacturer’s instructions. A media sample was taken from an acellular control gel at each timepoint and used for baseline correction. The standard protocol of the aggrecan Proteoglycan (PG) kit ELISA (Thermofisher—KAP1461) was followed.

#### 3.3.5. Compression Modulus

Gel dimensions were first measured, and gels were then placed on a stainless steel base plate in a Shimadzu Autograph AGS-X with a 1 kN load cell. The 15 mm diameter flat compression foot was lowered until it just began touching the gel. Gels were unsupported in the compression tests and allowed to deform laterally. The gel was pre-loaded to 0.05 N, and the displacement in mm was recorded. The gels were then loaded to 0.1 N, and the displacement was recorded. The range of 0.05–0.1 N was chosen as gel deformation was still linear and elastic within this range; however, tests were continued until total failure for each sample. The compression modulus (E) was then calculated from the following:

E = (F.h)/(A.d)

where F is the change in load (0.05 N), d is the recorded displacement between loads of 0.05 and 0.1 N, h is the height of the gel, and A is the cross-sectional area of the gel. Three samples were measured at each timepoint.

#### 3.3.6. Mass Retention

Gels were weighed immediately after printing and at days 3, 7, and 14 to assess the mass retention over this period.

### 3.4. Statistical Analysis

In all cases where a summary chart is presented, a two-way ANOVA with Tukey’s multiple comparison test were carried out on the data. The number of asterisks indicates the level of significance, with alpha equal to 0.05, i.e., *p* ≤ 0.05 is represented by *, *p* ≤ 0.01 is **, *p* ≤ 0.001 is ***, and *p* > 0.05 is not significant and not represented on the graph.

## 4. Conclusions

The ReJI bioprinting system allows for the production of scaffolds containing laminar co-cultures with a tight interface between the two phases of the co-culture. For an osteochondral co-culture, the IHC, ELISA, gel mass, and compression modulus results when taken together indicate that, for these specific co-cultures, scaffolds produced with high cell densities produce greater levels of ECM production and a more biomimetic in vitro culture than low-cell-density cultures. It is also clear that for the TC28a2 and SaoS-2 co-cultures, the presence of the other cell type further stimulates these cells for ECM production: in co-cultures with half of the chondrocytes and half of the osteoblasts (when compared to monocultures with the same cell density), higher levels of both bone and cartilage markers were produced. It is notable that the combination of having the cells in a co-culture and having a high cell density consistently had the highest levels of ECM production across a range of biochemical and mechanical assessment techniques. The results reinforce the need to generate scaffolds where cell–cell and cell–ECM interactions are enabled by having cells in close proximity to one another to rapidly produce native environments which give representative tissue models. The ReJI bioprinting approach used in this study offered a flexible and accurate process for this. Further work will consider a wider range of cell densities and substrates and the extended imaging of the morphology of the interface to explore how longer-term cultures can be used to create a representative interface for modelling disease development and drug effectiveness.

## Data Availability

The datasets generated for this study can be found in the Newcastle University Research Repository, https://doi.org/10.25405/data.ncl.24210762.v1.
